# The Design and Evaluation of Community‐Informed Video Resources to Promote Safe and Inclusive Cervical Screening for South Australian LGBTIQ+ People With a Cervix

**DOI:** 10.1002/hpja.70062

**Published:** 2025-06-22

**Authors:** Jennifer Baldock, Tamara Shipley, Victoria Paterson

**Affiliations:** ^1^ College of Medicine and Public Health Flinders University Bedford Park South Australia Australia; ^2^ Cancer Council SA Eastwood South Australia Australia; ^3^ SHINE SA Adelaide South Australia Australia

**Keywords:** cervical screening, gender diverse, prevention, transgender

## Abstract

**Issues Addressed:**

Lesbian, Gay, Bisexual, Transgender, Intersex, Queer or Questioning, and/or other people with a cervix (LGBTIQ+ people with a cervix) face barriers to cervical screening, leading to lower participation rates. Our study aimed to report on the design and evaluation of community‐informed video resources to promote safe and inclusive cervical screening for South Australian LGBTIQ+ people with a cervix.

**Methods:**

Two videos promoting inclusive cervical screening were developed by Cancer Council SA, SHINE SA and LGBTIQ+ people with a cervix for LGBTIQ+ people with a cervix (Video 1), and healthcare providers (Video 2). Evaluation involved two online surveys with LGBTIQ+ community members (*n* = 35) and healthcare providers (HCPs, *n* = 9) about their respective videos. Quantitative data were analysed descriptively in R; qualitative responses were thematically analysed using a general inductive approach.

**Results:**

The community‐focused video (Video 1) received positive feedback, with LGBTIQ+ people with a cervix feeling represented and expressing increased likelihood and intention to undergo screening. Responding to Video 2, half of the healthcare providers reported increased confidence in interacting with LGBTIQ+ people with a cervix and offering self‐collection.

**Conclusions:**

Improving cervical screening participation among LGBTIQ+ people with a cervix is essential to achieving the National Cervical Screening Program targets. This study highlights a respectful, community‐informed and relatively inexpensive approach to promote safe and inclusive cervical screening. The positive feedback from LGBTIQ+ people with a cervix underscores the value of inclusive messaging tailored to community needs.

**So What?:**

LGBTIQ+ people with a cervix face barriers to cervical screening participation, but self‐collection may alleviate some barriers. Community‐informed resources can effectively support this population to access inclusive cervical screening.

## 1 Introduction

Globally, cervical cancer, most commonly caused by human papillomavirus (HPV), is the fourth most common cancer in women [1]. One of the most significant risk factors for developing cervical cancer in countries with national screening programmes is being under‐screened or never‐screened for HPV [2]. Since the implementation of the National Cervical Screening Program (NCSP) in 1991, cervical cancer mortality in Australia has reduced by approximately 30% [3]. The current NCSP (updated in 2017) recommends that people with a cervix aged 25–74 complete a 5‐yearly HPV test [4].

In Australia, from 1 July 2022, people who are eligible for the NCSP have the option to ‘self‐collect’ a vaginal sample to test for HPV [5]. These self‐collected vaginal samples show comparable efficacy in detecting HPV, compared to clinician‐collected samples [5, 6].

To reach targets established in the ‘National Strategy for the Elimination of Cervical Cancer in Australia’ (NSECCA) it is necessary to increase cervical screening participation rates among vulnerable and under‐screened groups. This includes Lesbian, Gay, Bisexual, Transgender, Intersex, Queer or Questioning, and/or other people with a cervix (LGBTIQ+ people with a cervix) [7, 8].

Transgender and gender diverse (TGD) refer to people whose gender identity deviates from their assigned gender at birth or exists beyond the conventional binary understanding of gender. This may include people identifying as nonbinary, gender‐fluid, queer, gender‐nonconforming, and individuals identifying as men or women.^1^ As reported in the recently released National Action Plan for the Health and Wellbeing of LGBTIQ+ People 2025–2035 [9], these individuals experience significant disparities in cancer‐related health outcomes, including lower utilisation of population‐based cancer screening programs [8, 10, 11, 12].

Cervical screening is an invasive procedure and can be distressing for some. Barriers to cervical screening for LGBTIQ+ individuals include stigma, past negative experiences, fear of discrimination, lack of knowledge, misinformation, fear of penetration, trauma history and language or cultural obstacles [13, 14]. Furthermore, gendered assumptions and language^2^ used in health settings that are traditionally associated with cisgendered people (e.g., cervical screening settings such as a medical clinic) can lead to stress and/or avoidance [15], and the procedure itself can force TGD persons to confront parts of their bodies that they do not identify with, thereby increasing the risk of heightened dysphoria [16]. Self‐collection may be particularly appealing to under‐screened or never‐screened people, and LGBTIQ+ people with a cervix [17].

Recent Australian research has demonstrated that although many healthcare providers (HCPs) report feeling comfortable to treat LGBTIQ+ patients, they also report low levels of confidence and barriers to LGBTIQ+ service provision [18]. HCPs may have difficulty delivering confident, inclusive and culturally appropriate care due to a lack of knowledge, training, and resources specific to LGBTIQ+ populations [18, 19, 20].

Until relatively recently, Australian cervical screening messaging has largely overlooked LGBTIQ+ people with a cervix [16]. A recent systematic review revealed limited evidence of stronger outcomes from targeted interventions with cohorts of gender and sexuality diverse communities compared to general interventions, yet there was stronger evidence that these communities prefer targeted interventions [10].

Several Australian organisations, such as ACON (TransHub.org.au) [21] and Thorne Harbour Health (via Cancer Council Victoria) [22], have developed video resources promoting cervical screening and the self‐collection option, using community representatives. However, there is currently a gap in such resources specifically tailored for South Australians, featuring members of the South Australian community.

Our study aimed to report on the design and evaluation of community‐informed video resources to promote safe and inclusive cervical screening for South Australian LGBTIQ+ people with a cervix.

The specific objectives of this study were:

- To produce community‐informed video resources for community members and health professionals.
- To evaluate the video resources by gathering and describing responses from a sample of community members and health professionals.

## 2 Methods

Ethical approval was obtained for the evaluation methods from Cancer Council Victoria Human Research Ethics Committee. All participants provided informed consent prior to completing evaluation surveys. Documentation of all project components, including the logic model and budget allocations, is provided in Supporting Information 1.

### 2.1 Video Resources

Cancer Council SA partnered with SHINE SA and LGBTIQ+ people with a cervix to create two video resources aimed at promoting safe and inclusive cervical screening.

### 2.2 Recruitment of Community Representatives

SHINE SA conducted targeted outreach via email and community Facebook pages to invite community members to participate in the video project. Three community representatives were recruited and received a $250 grocery voucher in recognition of their time and contribution.

### 2.3 Resource Development Process

Prior to filming, community representatives participated in an online consultation meeting with SHINE SA to discuss video concepts and filming processes. This session provided an opportunity for community representatives to share lived experiences, ask questions, and inform the content direction.

Insights from this meeting were used to develop video scripts, which were reviewed by a nurse educator at SHINE SA, a TGD working group from a related project, and Cancer Council SA.

Community representatives were provided with the scripts and key prompts in advance. During filming, they were encouraged to speak in their own words and share personal stories where they felt comfortable, resulting in authentic and relatable content.

### 2.4 Evaluation Design

#### 2.4.1 Participants

The evaluation of this project involved two participant groups: LGBTIQ+ people with a cervix who are eligible for the NCSP (*n* = 35) and HCP (*n* = 9).

#### 2.4.2 Data Collection

Two online surveys were conducted using LimeSurvey, one targeting LGBTIQ+ community members with a cervix and the HCPs. In both surveys, participants viewed the resource video during survey completion. Questions assessing prior knowledge and existing practices (e.g., awareness of self‐collection, engagement with LGBTIQ+ patients) were asked before video viewing, while reactions to the video and intentions or confidence were captured post‐viewing. While the surveys did not use validated measures, all questions were developed in collaboration with SHINE SA staff to ensure clarity, relevance, and alignment with the project aims.

##### 2.4.2.1 Survey 1: Community Feedback

The community feedback survey (Supporting Information 2) was distributed through social media, SHINE SA's email lists, and at the FEAST Festival.

Specifically, the community feedback survey sought to:

1. Understand viewers' prior awareness of the self‐collection option.
2. Explore whether the video increased intention and likelihood to screen.
3. Assess the extent to which viewers felt represented in the video.
4. Gather feedback on how Cancer Council SA and SHINE SA can better support participation in cervical screening.

After completing the community feedback survey, participants received links to inclusive GP practices offering self‐collection.

##### 2.4.2.2 Survey 2: HCP Feedback

The HCP feedback survey (Supporting Information 3) was distributed through SHINE SA and Cancer Council SA's professional networks.

Specifically, the HCP feedback survey sought to:

1. Understand how frequently HCPs discuss cervical screening with the general population and LGBTIQ+ people with a cervix.
2. Assess whether the video increased HCPs' confidence in discussing and offering cervical screening and self‐collection to LGBTIQ+ people with a cervix.
3. Explore barriers to implementation and support needs for inclusive cervical screening.

After completing the HCP feedback survey, participants received information about seeking inclusion training.

### 2.5 Data Analysis

Graphics and descriptive analyses of quantitative data were completed using R (version 4.3.3) and RStudio (version 2023.03.0+386). Summary statistics are presented as frequencies and percentages. Qualitative data from the free‐text responses were thematically analysed using a general inductive approach, which enabled construction of key themes [23]. Themes were validated through discussion among authors.

## 3 Results

### 3.1 Video Resources

The first video was developed for LGBTIQ+ people with a cervix. It featured LGBTIQ+ people with a cervix sharing personal experiences and practical strategies to support a more comfortable and empowering cervical screening experience. The video also highlighted the self‐collection method and emphasised bodily autonomy and consent. For example:

So there's a lot of things you can do to make the experience more comfortable for you. You can bring a support person like a friend, a partner, or family member, you could use some kind of distraction like fidget toys, you can sit upright, you can ask to insert the speculum yourself—there's a lot of ways that you can feel empowered to make sure your test is your own and you're comfortable. (community representative)

Please refer to Supporting Information 4 to access the community‐focused video.

The second video was developed by the same group in collaboration with a nurse educator and was targeted at HCPs. It featured community representatives and the nurse educator discussing inclusive practices and communication. For example:

I think the best experience I've had was having a clinician just ask me upfront about my pronouns or how to refer to my body parts … because as she was going through the procedure, she was giving me a heads up of what she was about to do before doing a certain thing. (community representative)

This HCP‐focused video has been integrated into SHINE SA's Cervical Screening Training course. Please refer to Supporting Information 5 to access the HCP video.

### 3.2 Community Feedback

Thirty‐five LGBTIQ+ people with a cervix eligible for the NCSP responded to the community feedback survey. Participants' demographic characteristics, including gender identity, sexual orientation, transgender status and prior awareness of self‐collection cervical screening are summarised in Table 1. Just over half of participants were aware of the self‐collection option for cervical screening (54.3%, *n* = 19). Fewer than one‐quarter (22.85%, *n* = 8) indicated that a doctor had spoken to them about this option.

**TABLE 1:** Demographic characteristics of community feedback survey participants (*n* = 35).

Characteristic	*n*	%
Median age (IQR)	—	28 years (IQR: 12)
Gender identity		
Woman	16	45.7
Non‐binary	13	37.1
Man	6	17.1
Sexual orientation		
Bisexual	5	14.3
Gay	1	2.9
Heterosexual/straight	5	14.3
Lesbian	7	20.0
Pansexual	6	17.1
Queer	11	31.4
Transgender status		
Identifies as transgender	17	48.6
Questioning transgender status	2	5.7
Not transgender	14	40
Don't know	2	2.7
Awareness of cervical screen self‐collection method prior to video		
Aware	19	54.3
Not aware	16	45.7

#### 3.2.1 Representation of Thoughts and Feelings

**FIGURE 1:**
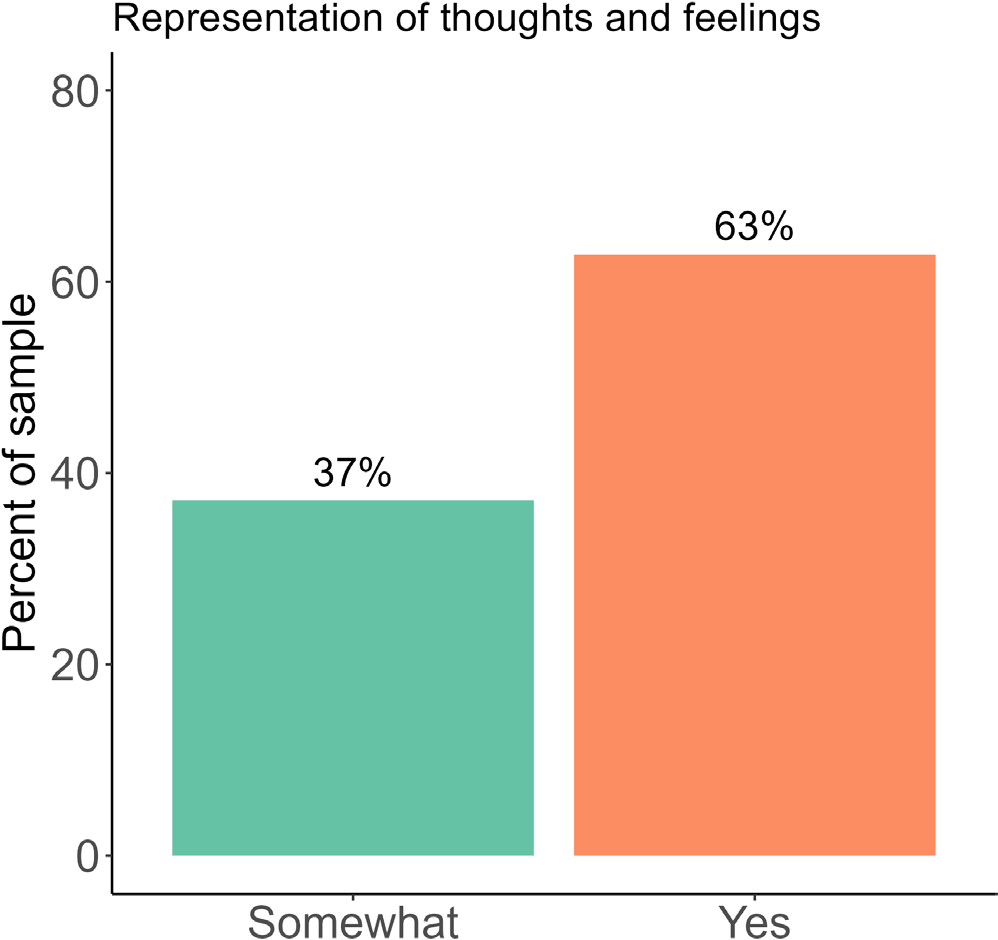
Responses regarding the video's representation of thoughts and feelings about cervical screening (*n* = 35).

All participants (100%) indicated that they thought the video at least somewhat represented their thoughts and feelings about cervical screening, with the majority indicating that it did (62.86%, *n =* 22; Figure 1). No participants shared any thoughts or feelings that were misrepresented or missing entirely in the video.

#### 3.2.2 Impact on Cervical Screening Participation

**FIGURE 2:**
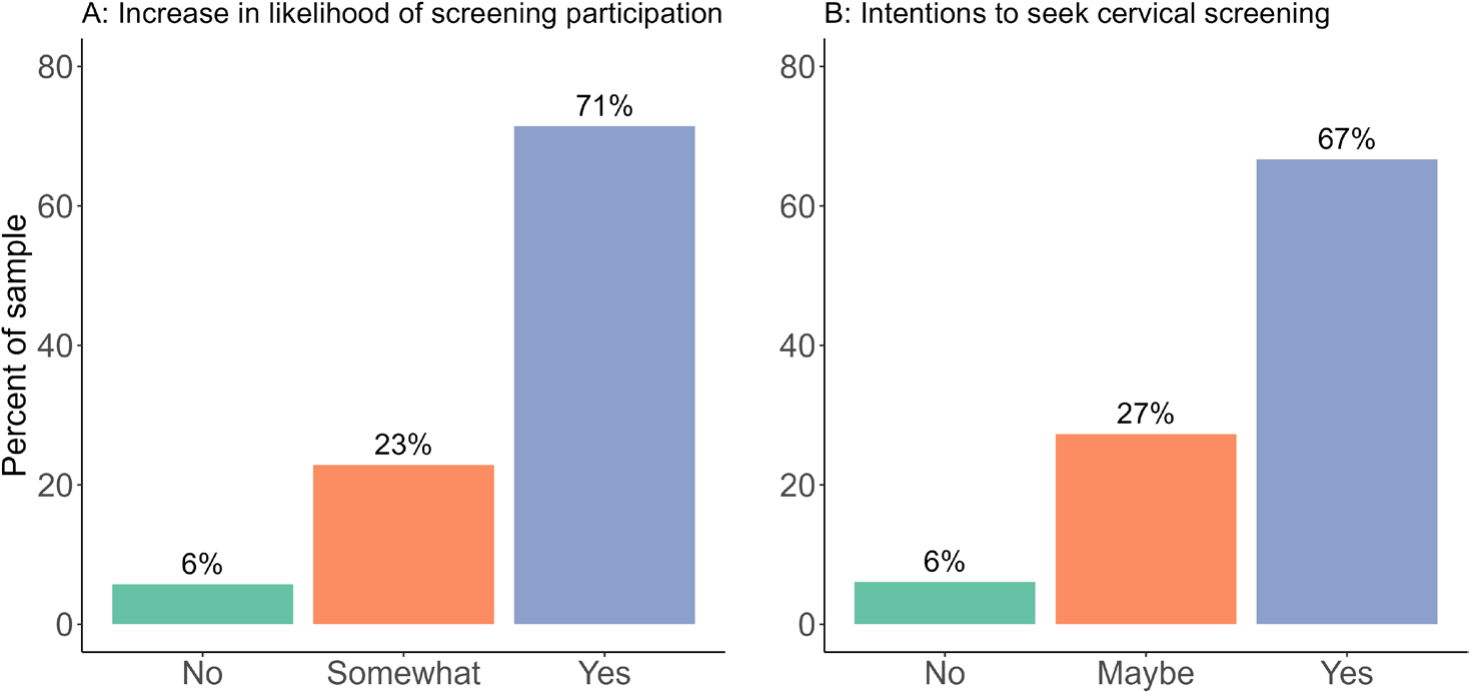
(A) The video's effectiveness in increasing participants' likelihood of completing cervical screening after video exposure (*n* = 35). (B) Intentions to seek out cervical screening after video exposure (*n* = 33, with two responses missing).

The majority (94%, *n =* 33) of participants indicated that the video at least somewhat increased the likelihood that they would complete a cervical screen (Figure 2A). The majority of participants also indicated that they intended to seek out cervical screening since seeing the video (66.67%, *n =* 22; Figure 2B).

#### 3.2.3 Community Member Support Needs for Cervical Screening Participation

Participants were asked to provide feedback on how Cancer Council SA and SHINE SA could better support them in participating in cervical screening. From the comments received, two main themes were constructed: accessibility and information.

Accessibility was highlighted by several respondents who expressed the need for easier appointment scheduling, suggesting the desire for options such as availability outside of typical 9am – 5pm timefrane. Some also mentioned the importance of having a list of GP clinics offering self‐collection, although it was noted that this information was provided as part of the survey. Additionally, one respondent said that they would appreciate knowing whether HCPs administering the screening had experience with or were themselves transgender individuals, for added reassurance.

The second theme focused on information. Many respondents simply appreciated knowing about the availability of self‐collection. Suggestions were made to circulate the community‐focused video more widely, and there was a request for clearer information on what cervical screening tests for.

Additional feedback highlighted positive reactions from community members regarding the representation of diversity in the video. For example, one respondent remarked ‘It was great to see representation of lots of different types of people and different body types and identities’. Another specifically praised the inclusion of local community members. One respondent commended the video for being ‘…very concise and easy to understand’ and emphasised the importance of seeing gender‐diverse individuals conveying the information.

One respondent expressed that they found it ‘really comforting to see people feeling the same way I do about [cervical screening]’, while another comment related to the video production which relied on auto‐generated captions provided by YouTube. One respondent suggested that ‘having proper subtitles included would be fantastic’.

In general, community members found the community‐focused video to be clear, helpful, respectful, and inclusive. Interest was expressed for sharing more health information in this format.

### 3.3 HCP Feedback

#### 3.3.1 HCPs Offering Self‐Collection

Nine HCPs responded to their feedback survey. All responding HCPs indicated that they offered the self‐collection option for cervical screening in their clinics^3^ and none had any reservations about doing so. However, of the sample, only 22.22% (*n* = 2) indicated that their practice was registered on Preventive Health SA's list of clinics offering self‐collection cervical screening.

#### 3.3.2 Discussion of Cervical Screening With Patients

**FIGURE 3:**
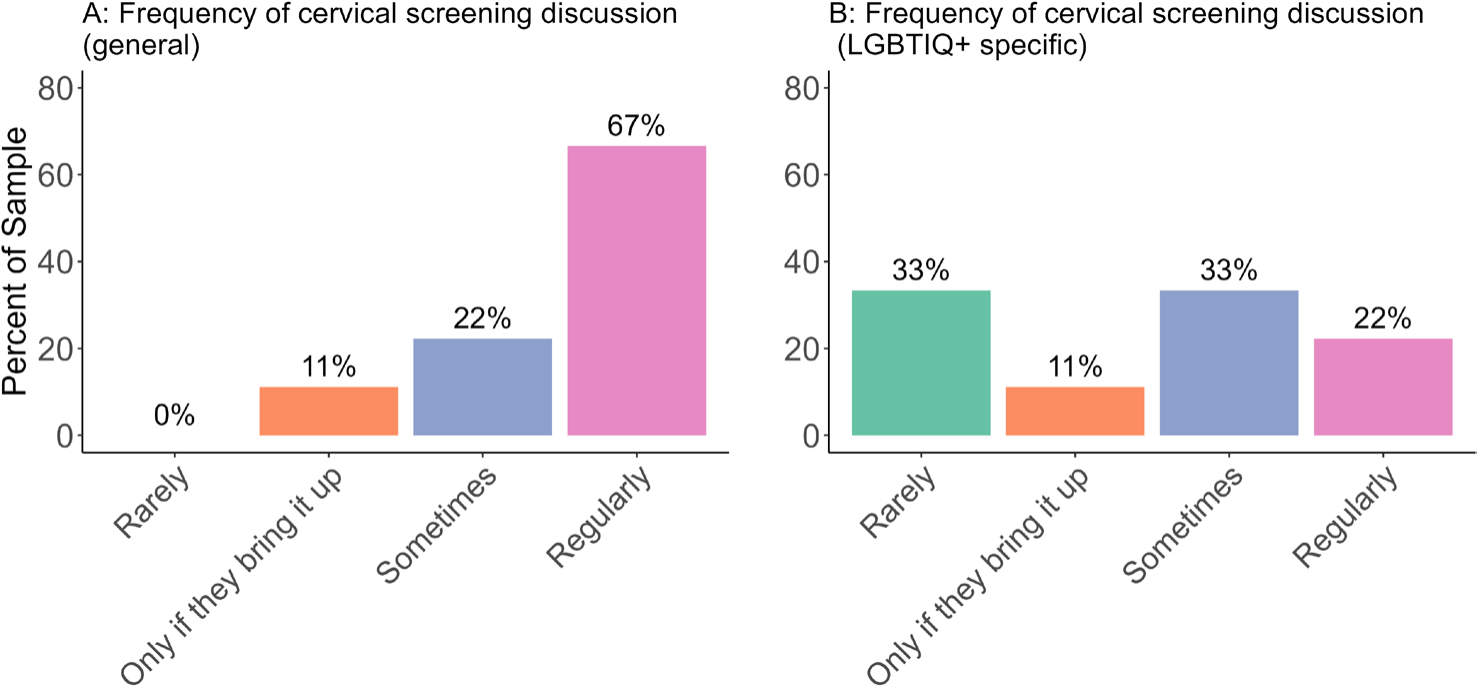
(A) Self‐reported frequency of HCP' cervical screening discussion with the general population patients, compared to LGBTIQ+ specific patients (B).

Of the sample, 66.67% (*n =* 6) of the HCPs reported that they regularly discuss cervical screening with their patients (Figure 3A). However, only 22.22% (*n =* 2) indicated that they regularly discuss cervical screening with LGBTIQ+ people with a cervix, specifically (Figure 3B), highlighting a discrepancy.

#### 3.3.3 HCP Video's Effect on HCP Confidence

**FIGURE 4:**
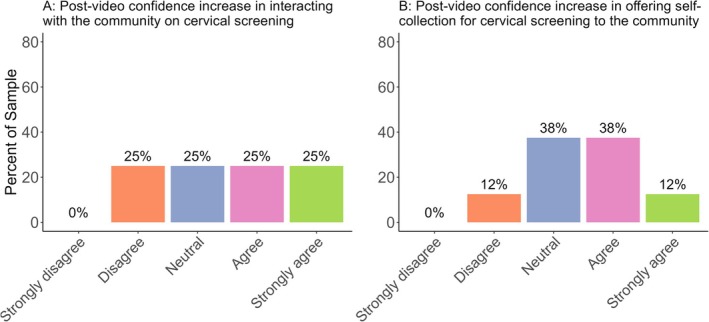
(A) Self‐reported increase in confidence in interacting with the LGBTIQ+ community on self‐collection cervical screening option after watching the video. (B) Self‐reported increase in confidence offering the self‐collection cervical screening option.

HCPs (*n =* 8)^4^ were asked to rate their level of agreement with the statement that the video resource made them feel more confident in interacting with the LGBTIQ+ people with a cervix regarding cervical screening. Half of the respondents either agreed or strongly agreed with this statement (*n =* 4; Figure 4A). One HCP indicated the video seemed to be focused more on ‘advertising/suggesting for attending inclusion training’ as opposed to enhancing confidence.

Similarly, HCPs were asked to rate their level of agreement with the statement that the video resource made them feel more confident in offering the self‐collection cervical screening option to LGBTIQ+ people with a cervix. Again, half of the respondents either agreed or strongly agreed with this statement (*n* = 4; Figure 4B).

#### 3.3.4 HCP Support Needs for Inclusive Cervical Screening

HCPs were also asked to report any barriers to offering self‐collected cervical screening provision. One HCP cited time constraints as a significant barrier to offering self‐collection. Another pointed out the challenge of offering the self‐collection option to people with lower literacy levels, suggesting the use of photo demonstrations to improve comprehension. However, they also expressed concern about not being able to tell if a patient had completed the collection correctly. Fear and apprehension were mentioned by one HCP as barriers to discussing cervical screening with eligible patients.

It was suggested that disseminating resources promoting the importance of screening within the community could encourage individuals to pursue screening and consider self‐collection. One HCP found the video resource helpful, despite not facing any significant barriers themselves. They stressed the ongoing need to develop valuable resources like the video developed in this project.

When asked about how Cancer Council SA could better support HCPs in this area, recommendations included promotion of the importance of screening through various channels, including advertising and community‐specific materials. Additionally, they suggested providing inclusivity training to HCPs. Another HCP emphasised the importance of collaborating with HCPs to co‐design resources directed at the various professions. Such resources were seen as essential in addressing barriers and supporting cervical screening initiatives in under‐screened populations.

## 4 Discussion

This project developed and evaluated video resources to promote safe and inclusive cervical screening for South Australian LGBTIQ+ people with a cervix.

### 4.1 Community Members

Fewer than one‐quarter of community members reported that their doctor had discussed the option of self‐collection for cervical screening with them. Two‐thirds HCPs reported discussing cervical screening with patients in general, but fewer than one‐quarter reported discussing it specifically with LGBTIQ+ people with a cervix. These findings supplement recent results from a larger Australian study, which found that only 32.7% of TGD participants had discussed cervical cancer with a HCP, and less than half had been recommended cervical screening [24].

This disparity could be due to HCPs not recognising that some of the men or non‐binary people they encounter are eligible for cervical screening [16], or, as was noted by one HCP in the current evaluation, they also might be hesitant to discuss cervical screening with LGBTIQ+ people with a cervix for fear of causing discomfort, as has been found in other studies [25]. This highlights a need for future research and service development ensuring HCPs possess the knowledge, skills, and competency to communicate effectively with LGBTIQ+ people with a cervix about cervical screening [26].

The developed video resources address a significant gap in cervical screening materials by representing LGBTIQ+ South Australians with a cervix, whose bodies and experiences have often been excluded from cancer prevention discourse [16]. Evaluation results showed that community members felt seen and heard, with their thoughts and feelings reflected in the community‐focused video. This is a crucial step in ensuring the message is relevant and meets the needs of the intended audience [27].

Most community members reported that the video had increased the likelihood that they would complete cervical screening (either self‐collection or clinician collected) and that they intend to seek out cervical screening since seeing the video. These findings highlight the acceptability and potential effectiveness of targeted video resources that prioritise community representation in communicating health messaging, in line with findings from Drysdale et al. suggesting that LGBTIQ+ people prefer targeted interventions [10].

Despite this, the extent to which increased likelihood and intention to screen translates into actual cervical screening behaviour remains unknown. While intention can be a key predictor of behaviour [28], evidence shows that intention–behaviour gaps are common, especially among marginalised populations who experience barriers to healthcare access [29]. To determine whether the video resources developed in this project result in increases in cervical screening participation, more comprehensive behaviour change evaluations are required. For example, triangulating self‐reported intention data with service‐level screening records (where available) and incorporating qualitative insights and pre and post measures would provide a more comprehensive assessment of project outcomes.

The qualitative community feedback highlighted accessibility needs encompassing both physical access and the inclusivity of such services for diverse communities. The need for clear, culturally appropriate, and readily available resources regarding cervical screening options was also reaffirmed by participants, suggesting a current dearth of this information in the current healthcare landscape.

In response to community feedback, all resource videos were updated to include subtitles to improve accessibility and information delivery. Subtitling is a well‐established strategy to enhance comprehension and engagement among all audiences (e.g., hearing impaired, low literacy) [30]. However, accessibility requires consideration beyond subtitles and includes availability, timing, and cost of attending preventive health appointments.

Community feedback on the videos reflects broader evidence reported in the recently released National Action Plan for the Health and Wellbeing of LGBTIQ+ People 2025–2035, which underscores the importance of inclusive and representative cancer screening messaging to address disparities and improve relevance, trust, and uptake of preventive care services among LGBTIQ+ communities [9]. Integral to this is the need to address additional, well‐documented barriers experienced by LGBTIQ+ people with a cervix, such as limited HCP knowledge and stigmatising attitudes [20].

### 4.2 HCPs

Ussher et al. [18] reported that HCPs report low levels of confidence and knowledge regarding LGBTIQ+ service provision. Thus, a focus of the evaluation was to assess the impact of the video on HCP confidence. Of the eight participating HCPs who viewed the video, half reported an increase in confidence regarding interacting with LGBTIQ+ people with a cervix about cervical screening, and half reported increased confidence in offering the self‐collection option to eligible LGBTIQ+ people with a cervix.

One HCP noted that they perceived the video's focus to be more on promoting inclusion training rather than enhancing confidence. This feedback was incorporated during video editing to ensure the final video had more direct advice for HCPs, while acknowledging that LGBTIQ+ inclusion training could be a point of action.

Further research is needed to assess how the HCP‐focused video may influence provider confidence, ideally using validated measures and pre‐ and post‐assessment designs. In this study, the small sample size and use of unvalidated tools limited the generalisability of findings. Future efforts would benefit from involving a broader group of HCPs in the video design process and engaging them more actively in shaping the evaluation approach.

HCPs noted that they need information provided in a way that will reduce time burden. This may include HCP training and additional resources used for explaining the test to various types of people (e.g., LGBTIQ+, low literacy, culturally and linguistically diverse, etc.). Additional training may also alleviate apprehension that some HCPs may experience when interacting with LGBTIQ+ people with a cervix regarding cervical screening [18, 25]. More research is needed to better understand the unmet learning and resourcing needs of HCPs offering cervical screening, and future interventions should be co‐designed with HCPs to ensure they are practically feasible for clinical settings and that they reduce time burden rather than add to it.

### 4.3 Strengths and Limitations

A key strength of this project was its multi‐organisational collaboration. SHINE SA, with its long‐standing relationship with the LGBTIQ+ community, served as a trusted bridge between Cancer Council SA and the community. They provided a safe and inclusive environment for recruiting community representatives, supported the filming process, and guided the development of key messages to ensure cultural relevance and inclusivity. Evaluation findings highlighted the value of strong local representation, which was largely enabled by this collaborative approach.

While this project prioritised local community representation, the information conveyed in the videos only reflected the personal views of those who were featured, which may be viewed as a limitation. This was done intentionally, to ensure the videos portrayed authentic experiences. However, future resource development could aim to include a broader range of voices and perspectives, ensuring the community has access to a more diverse suite of viewpoints and experiences.

Evaluation findings suggest that the level of engagement used was acceptable for designing and disseminating cervical screening video resources for LGBTIQ+ people with a cervix and HCPs. However, community consultation was limited to the resource development phase only. As such, the project did not adopt a full community‐based participatory approach.

Participatory design entails involving multiple community members in all stages of the project, from conceptualisation and development to dissemination, evaluation design, and ensuring project sustainability [31]. While these methods require significant time and resources to execute effectively, research indicates that such methods are particularly effective in developing health information for LGBTIQ+ people e.g., [27, 31, 32]. Therefore, it may be advisable to employ these methods in future initiatives.

Since this project's completion, Preventive Health SA separately funded SHINE SA to engage the TGD community, conduct targeted screening clinics outside regular hours (i.e., 9am – 5pm), offer training for non‐health professionals, update GP resources, and collaborate on LGBTIQ+ health promotion. The current evaluation results affirm these subsequent initiatives, validating the needs identified in the current evaluation for enhanced service accessibility, resources, and provider training. Additional future directions can be found in Box 1.

BOX 1 **Future directions and alignment with the National Action Plan for the Health and Wellbeing of LGBTIQ+ People 2025–2035 [9].**

- Assess the feasibility of a mailed self‐collection model, like the National Bowel Cancer Screening Program, in which eligible individuals receive a free kit by mail and return it via prepaid envelope. This recommendation responds to community feedback highlighting the need for more accessible cervical screening options and aligns with Action 4C: Explore new and more flexible approaches, including adapting existing resources for screening, to make screening for cancer and other diseases more accessible for LGBTIQA+ people, with a focus on areas where they are often under‐screened.
- Undertake further research to understand the needs of health care professionals in delivering safe and inclusive cervical screening. This stems from limited responses to the health professional survey and feedback calling for co‐designed resources to support inclusive care and aligns with Action 7: Build capacity and scale of health and wellbeing services for LGBTIQA+ people.
- Strengthen engagement with LGBTIQ+ people with a cervix through community‐based participatory approaches to guide the scale‐up of the project in larger jurisdictions. This recommendation reflects community calls for more authentic and sustained involvement in both the design and research phases and aligns with Action 5: Foster community connections within and across LGBTIQA+ communities.

## 5 Conclusion

Cervical screening participation among LGBTIQ+ people with a cervix must increase to meet targets set out in the NSECCA. Therefore, it is essential to effectively communicate updated cervical screening messaging to LGBTIQ+ people with a cervix as the self‐collection option offers a convenient and less invasive alternative to traditional methods, especially for those facing barriers to screening. This project represents an example of a relatively inexpensive method for engagement and resource production that can be used to promote cervical screening to LGBTIQ+ people with a cervix in a respectful and inclusive way.

## Conflicts of Interest

The authors declare no conflicts of interest.

## Supporting information


**Data S1.** Supporting Information.

**Data S2.** Supporting Information.

**Data S3.** Supporting Information.

**Data S4.** Supporting Information.

**Data S5.** Supporting Information.

## Data Availability

The data that support the findings of this study are available on request from the corresponding author. The data are not publicly available due to privacy or ethical restrictions.

## Endnotes

^1^
We acknowledge that individuals may identify with one or more sexualities and gender identities within the LGBTIQ+ spectrum. Therefore, the term ‘LGBTIQ+ people with a cervix’ (rather than just TDG) is used to ensure inclusivity of the diverse identities that this project is applicable for.

^2^
An example of gendered language around cervical screening can be seen in the first line of this article. The World Health Organization's resource on cervical cancer only refers to ‘women’ in relation to cervical cancer. This kind of cisnormativity further marginalises LGBTIQ+ people with a cervix who do not identify with that gender label.

^3^
One HCP did not answer this question.

^4^
One HCP was unable to view the video and thus, their responses were excluded from the post‐video questions.
