# Supplementary material for: The Design and Evaluation of Community‐Informed Video Resources to Promote Safe and Inclusive Cervical Screening for South Australian LGBTIQ+ People With a Cervix
Source: Health Promot J Austr. 2025 Jun 22;36(3):e70062. doi: 10.1002/hpja.70062 (PMC12183492; doi:10.1002/hpja.70062)
Supplement: Supplementary file 1 — Data S1. Supporting Information. [file HPJA-36-0-s005.docx]

# Supporting Information 1: Summary of the project components

# Legend: Summary of project components, word document.

Evidence underscores the need to support LGBTIQ+ communities in cervical screening by addressing both individual-level factors (e.g., lack of knowledge and LGBTIQ+-specific information) and system-level factors (e.g., safe, culturally competent care). Barriers at both levels highlight the importance of targeted interventions that empower LGBTIQ+ and equip HCPs. This project, collaboratively designed by Cancer Council SA and Shine SA, aimed to address these issues by co-designing inclusive cervical screening resources for LGBTIQ+ people (individual-level) and HCPs (system-level) with a focus on community representation. Selected members of the cancer care workforce also engaged in inclusivity training as part of this project (system-level). The projects logic model can be found in Figure 1.


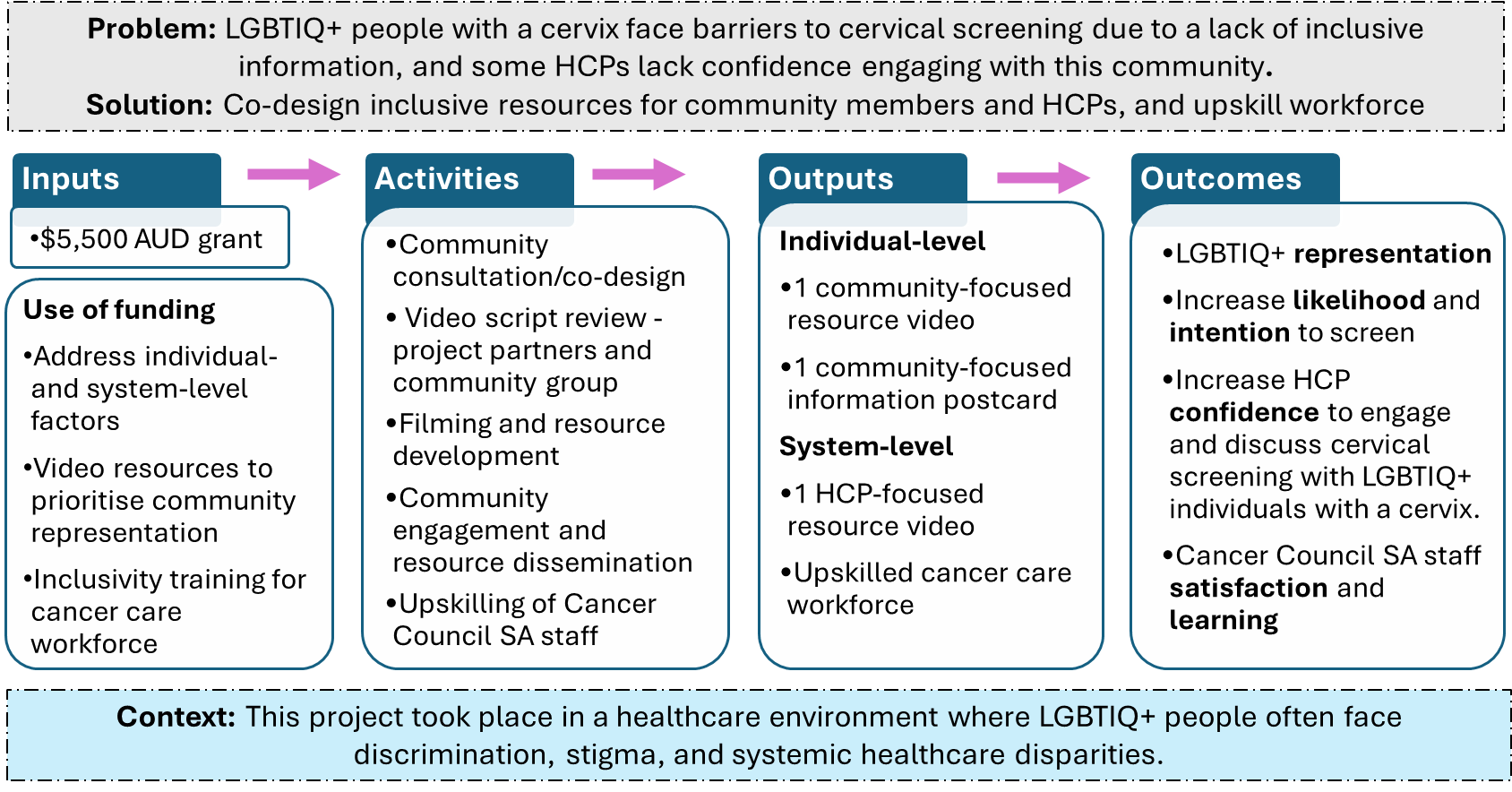


### Budget

This project was funded by a $5,500 AUD grant from [REDACTED]. The funding was primarily allocated to areas including resource production, community engagement, upskilling of Cancer Council SA staff, and participant compensation.

To ensure maximum cost-effectiveness, we capitalised on existing resources within Cancer Council SA, including personnel expertise and infrastructure. Time dedicated to developing and disseminating the resources, and conducting the evaluation was part of staff's existing duties, so these costs were not included in the budget. Furthermore, we opted for free or low-cost tools and platforms for data collection and analysis wherever possible. For a detailed breakdown of grant spending allocations, please refer to Table 1.

| **Description** | **Cost (exc GST)** |
| --- | --- |
| LGBTIQ inclusion workshop for 4 Cancer Council SA nurses and 2 Cancer Council SA social workers | $392.76 |
| Foundations of LGBTIQ+ Inclusion workshops for 6 Cancer Council SA staff | $327.30 |
| Video shoot for 2 videos | $2,262.15 |
| Printing postcard resource | $376.00 |
| Gift cards for video talent (3x$250) | $763.90 |
| Gift cards for evaluation prize draw (2x$200) | $400.00 |
| Branded tote bags to give away at Feast Festival | $725.00 |
| 5 branded shirts for Feast Festival | $223.34 |
| Pride inclusion flag and pin | $29.55 |
| TOTAL | $5,500 |

### Upskilling cancer care workforce:

A portion of the grant funding was allocated to upskilling Cancer Council SA staff, including nurses, social workers, prevention officers, researchers, marketing and communications personnel and human resources officers, through participation in SHINE SA's ‘Foundations of LGBTIQ+ Inclusion’ workshops. Staff were encouraged to engage in peer-to-peer learning to distribute knowledge throughout the organisation.

Cancer Council SA staff who participated in the ‘Foundations of LGBTIQ+ Inclusion’ workshops received an online post-workshop survey. A survey collected quantitative feedback on workshop aspects such as content relevance, learning, and overall satisfaction.

All responding staff members (*n*=7) agreed that the workshops were beneficial (100%), stating that they acquired new knowledge (100%). Furthermore, they expressed a willingness to recommend the workshops to others (100%). The majority of responding staff members (85.7%) indicated that they did not find the content confusing and that the workshop covered the content that they expected (71.4%).

The survey sought insights from staff members regarding their approaches to sharing workshop learnings with their respective teams. While one staff member acknowledged they had not yet widely disseminated the information, another actively integrated new knowledge into their communication strategies. Additionally, two staff members summarised the workshop content during their team meetings.

Cancer Council SA staff were satisfied with the inclusion workshop and engaged in peer-to-peer information sharing.
