# Supplementary material for: The Design and Evaluation of Community‐Informed Video Resources to Promote Safe and Inclusive Cervical Screening for South Australian LGBTIQ+ People With a Cervix
Source: Health Promot J Austr. 2025 Jun 22;36(3):e70062. doi: 10.1002/hpja.70062 (PMC12183492; doi:10.1002/hpja.70062)
Supplement: Supplementary file 2 — Data S2. Supporting Information. [file HPJA-36-0-s002.docx]

# Supporting Information 2: Community feedback survey

Legend: Data collection survey, word document.

**Consent**

Are you at least 18 years of age and do you consent to take part in this survey? Please note: This survey is voluntary and the information you provide will be treated with confidence. Your data will be anonymous. Only completed responses will be retained as incomplete responses will be treated as a withdrawal of participation. If you wish to withdraw, simply close the window, and do not attempt the survey again. If you choose to leave your contact number at the end of the survey for entry into the prize draw, you consent to be contacted via this phone number for the purposes of the prize draw. You can withdraw your participation after you complete the survey, if you have left a contact number and contact the research team REDACTED prior to December 8th, 2023:

Please choose only one of the following:

• Yes

• No

**Do you have a cervix? ***

Please choose only one of the following:

• Yes

• No

• Don't know

• Rather not say

**What is your age? (must be 18+) ***

Your answer must be at least 18

Please write your answer here:

•

**How do you identify? ***

Please choose only one of the following:

• Woman

• Man

• Non-binary

• Questioning

• Rather not say

• Other

**What is your sexual orientation? ***

Please choose only one of the following:

• Asexual

• Bisexual

• Heterosexual/straight

• Lesbian

• Gay

• Pansexual

• Queer

• Questioning

• Rather not say

• Other

**Do you consider yourself to be transgender or gender diverse? ***

Please choose only one of the following:

• Yes

• No

• Questioning

• Don't know

• Rather not say

Please watch this video before answering the following questions

**Prior to this video, were you aware of the cervical screening self-collection option? ***

Please choose only one of the following:

• Yes

• No

• Don't know

**Has a doctor ever spoken to you about the cervical screening self collection option? ***

Please choose only one of the following:

• Yes

• No

• Not applicable

Make a comment on your choice here:

**Did this video increase the likelihood that you would complete a cervical screen (whether self-screen or health professional screen) ***

Please choose only one of the following:

• Yes

• Somewhat

• No

• Not applicable

Make a comment on your choice here:

**Are your thoughts/feelings about cervical screening represented in this video? ***

Please choose only one of the following:

• Yes

• Somewhat

• No

• Not applicable

**Would you like to share any thoughts/feelings that were unrepresented or misrepresented in this video?**

Please write your answer here:

**How can Cancer Council SA and Shine best support you in participating in cervical screening?**

Please write your answer here:

**Do you intend to seek out cervical screening?**

Please choose only one of the following:

• Yes

• Maybe

• No

• Not applicable

**Do you have any general feedback about the video?**

Please write your answer here:

**To be entered into the prize draw to win a $200 Coles groceries gift card (cannot be used to purchase alcohol or cigarettes) please leave your phone number**

Please write your answer here:

**If you would like a list of GP clinics that offer self-collection, you can find that here: http://www.wellbeingsa.sa.gov.au/your-wellbeing/your-health-checks-screening/cervical-screening/self-collection-providers**

**If you would like a list of GP clinics that are safe and inclusive for trans and gender-diverse people, you can find that here: https://transhealthsa.com/directory/**

**If you would like to view the video resources or show a friend, you can find that here: http://www.youtube.com/watch?v=OZSv18fN9VA**

**If you would like to share this survey with other people, please use the following link: http://survey.cancersa.org.au/index.php/771171?lang=en**

**If any aspect of completing this survey caused you distress, you can contact the Cancer Council Helpline on 13 11 20. If you need immediate assistance, please contact Lifeline 24-hour crisis support on 13 11 14.**

**For further information on cervical cancer, cervical screening, and self-collection please visit: https://www.cancersa.org.au/prevention/finding-cancer-early/finding-cervical-cancer-early/**
