# Supplementary material for: The Design and Evaluation of Community‐Informed Video Resources to Promote Safe and Inclusive Cervical Screening for South Australian LGBTIQ+ People With a Cervix
Source: Health Promot J Austr. 2025 Jun 22;36(3):e70062. doi: 10.1002/hpja.70062 (PMC12183492; doi:10.1002/hpja.70062)
Supplement: Supplementary file 3 — Data S3. Supporting Information. [file HPJA-36-0-s001.docx]

# Supporting information 3: Healthcare provider feedback survey

Legend: Data collection survey, word document.

**Consent**

Are you at least 18 years of age and do you consent to take part in this survey? Please note: This survey is voluntary and the information you provide will be treated with confidence. Your data will be anonymous. Only completed responses will be retained as incomplete responses will be treated as a withdrawal of participation. If you wish to withdraw, simply close the window, and do not attempt the survey again. If you choose to leave your contact number at the end of the survey for entry into the prize draw, you consent to be contacted via this phone number for the purposes of the prize draw. You can withdraw your participation after you complete the survey, if you have left a contact number and contact the research team REDACTED prior to December 8th, 2023: *

Please choose only one of the following:

• Yes

• No

**How often would you discuss cervical screening with your patients in general? ***

Please choose only one of the following:

• Regularly

• Sometimes

• Rarely

• Only if they bring it up

• Not sure/don't know

• I do not perform cervical screenings as part of my routine care

Make a comment on your choice here:

**How often would you discuss cervical screening with people with a cervix who are part of the LGBTQI+ community (e.g. trans men, non-binary, intersex etc) ***

Please choose only one of the following:

• Regularly

• Sometimes

• Rarely

• Only if they bring it up

• Not sure/don't know

• I do not perform cervical screening as part of my routine care

Make a comment on your choice here:

Please watch this video before answering the following questions

**Did the video resource make you feel more confident in interacting with people with a cervix who are part of the LGBTIQ+ community (e.g. trans men, non-binary, intersex, gender diverse etc) about cervical screening?** *

Please choose only one of the following:

• Strongly agree

• Agree

• Neutral

• Disagree

• Strongly disagree

Make a comment on your choice here:

**Did the video make you feel more confident in offering cervical screening self-collection to people with a cervix who are part of the LGBTIQ+ community (e.g. trans men, non-binary, intersex, gender diverse etc.) about cervical screening? ***

Please choose only one of the following:

• Strongly agree

• Agree

• Neutral

• Disagree

• Strongly disagree

Make a comment on your choice here:

**Does your practice offer self-collection cervical screening?**

Please choose only one of the following:

• Yes

• No

• Don't know

**Do you have plans to implement the cervical screening self-collection option for all people with a cervix? ***

Please choose only one of the following:

• Yes

• No

• Don't know

Make a comment on your choice here:

**Do you have any reservations about providing cervical screening self-collection option to all people with a cervix? ***

Please write your answer here:

**What are (if any) the barriers to providing cervical screening self-collection option? ***

Please write your answer here:

**How can Cancer Council SA and Shine best support you in providing cervical screening to people with a cervix who are part of the LGBTIQ+ community? ***

Please write your answer here:

**Is your practice registered on Wellbeing SA's list of providers offering self-collection cervical screening? You can register to be part of that list by emailing wellbeingsacancerscreening@sa.gov.au. ***

Please choose only one of the following:

• Yes

• No

• Don't know

**Please contact SHINE SA at https://shinesa.org.au/ if you would like to engage in LGBTIQ+ inclusion training**

**To be entered into the prize fraw to win a $200 Coles groceries gift card (cannot be used to purchase alcohol or cigarettes), please leave your phone number:**

Please write your answer here:
