# Supplementary material for: The Design and Evaluation of Community‐Informed Video Resources to Promote Safe and Inclusive Cervical Screening for South Australian LGBTIQ+ People With a Cervix
Source: Health Promot J Austr. 2025 Jun 22;36(3):e70062. doi: 10.1002/hpja.70062 (PMC12183492; doi:10.1002/hpja.70062)
Supplement: Supplementary file 4 — Data S4. Supporting Information. [file HPJA-36-0-s004.docx]

# Supporting Information 4: HCP video

<https://youtu.be/O-KectlTh4s>

Legend: Link to healthcare providers video, word document
