# Supplementary material for: The Design and Evaluation of Community‐Informed Video Resources to Promote Safe and Inclusive Cervical Screening for South Australian LGBTIQ+ People With a Cervix
Source: Health Promot J Austr. 2025 Jun 22;36(3):e70062. doi: 10.1002/hpja.70062 (PMC12183492; doi:10.1002/hpja.70062)
Supplement: Supplementary file 5 — Data S5. Supporting Information. [file HPJA-36-0-s003.docx]

# Supporting Information 5: HCP video

[https://youtu.be/HD9iu7o8dOg?si=zFzrf6BY1iUmQUfY](https://aus01.safelinks.protection.outlook.com/?url=https%3A%2F%2Fyoutu.be%2FHD9iu7o8dOg%3Fsi%3DzFzrf6BY1iUmQUfY&data=05%7C02%7Cjbaldock%40cancersa.org.au%7Cf48ae71a80ba4510e8bb08dced7fc325%7C9d3aea62e4d946acbcee7c3e0e63ec42%7C0%7C0%7C638646380265373850%7CUnknown%7CTWFpbGZsb3d8eyJWIjoiMC4wLjAwMDAiLCJQIjoiV2luMzIiLCJBTiI6Ik1haWwiLCJXVCI6Mn0%3D%7C0%7C%7C%7C&sdata=G2uyiNN2IiPtkNPcaKeHHx7BTWOodXAXWFHl5zvQL%2BU%3D&reserved=0) Legend: Link to healthcare providers video, word document
